# The Perils of Excessively Relying on Medicine’s Tradition of Standardization

**DOI:** 10.5334/pme.1864

**Published:** 2025-07-02

**Authors:** Lara Varpio, Daniel J. Schumacher, Sayra M. Cristancho

**Affiliations:** 1 Department of Pediatrics, Perelman School of Medicine, University of Pennsylvania, US; 2 Medical Education Collaboratory, The Children’s Hospital of Philadelphia, US; 3 Department of Pediatrics, Cincinnati Children’s Hospital Medical Center/University of Cincinnati College of Medicine, Cincinnati, Ohio, US; 4 Department of Surgery and Department of Anatomy and Cell Biology, and Scientist, Centre for Education, Research, and Innovation (CERI), Schulich School of Medicine & Dentistry, Western University, CA

## Abstract

Medicine has long relied on standardization to ensure safety, consistency, and efficiency. From evidence-based guidelines to competency-based curricula, standardized practices have shaped both clinical care and medical education. Yet, as social conditions evolve and clinical complexity increases, this commentary argues that rigid adherence to standardized protocols can become a liability. We explore how excessive standardization can constrain expert judgment, perpetuate inequities in education, and hinder responsiveness to emergent societal needs. Drawing on examples from admissions, assessment, and clinical practice, we show how the very structures meant to promote fairness and safety can inadvertently undermine equity and adaptability. We call for a shift toward “adaptive standardization”—an approach that balances consistency with contextual flexibility. Such a shift requires not only individual discernment but also systemic support for clinicians and educators to tailor decisions to specific circumstances. Ultimately, we argue that fostering adaptability alongside standardization is essential for medical systems to remain responsive, just, and resilient in a rapidly changing world.

## Introduction

*Perspective on Medical Education’s* pages are regularly populated with calls for physicians to contend with social change, urging recognition of health professions education-related implications for developments like global warming, legislation changes, and public distrust in science. But contending with these developments is daunting; it requires change. And before embarking on the drudgery of change, we want evidence that change is required. Indeed, unless we know that *it’s broke*, why would we try to *fix it*? But identifying what needs changing can be problematic when the *thing to be changed* isn’t readily quantifiable or tangible. Sometimes what needs changing is an aspect of the medical profession’s very foundation. In this commentary, we address one such foundational element. We propose that contending with the rapid change of social developments requires relinquishing overreliance on *standardization* and leaning into the affordances of *adaptability*.

## In defense of standardization

To be sure, standardization has greatly contributed to health care. For example, standardization—defined as “a process of constructing uniformities across time and space, through the generation of agreed-upon rules” [1]—is foundational to evidence-based medicine (EBM). EBM strives to establish research evidence as the basis for clinical decision making and, in so doing, to reduce variation, to increase efficiency, and to bolster patient safety [2,3]. In support of this goal, clinical practice guidelines (CPGs) are created to transform available evidence about a specific condition into “standardized, actionable information to guide practice.” [4] The standardization realized in CPGs helps to ensure that patients receive appropriate care. Perhaps best known are pre-procedure timeouts that prevent wrong person, wrong site, and wrong procedure operations [5] and checklists for preventing central-line associated bloodstream infections [6]. Many other examples exist, many within specific specialties, such as the Pediatric Respiratory Assessment Measure that can be used to assess asthma severity and direct initial treatment which can be started by nurses or respiratory therapists. Clearly, standardization can help reap positive patient outcomes.

Standardization is also foundational to medical education—across the undergraduate (i.e., medical student), graduate (i.e., resident and fellow), and continuing (i.e., continuing professional development) education levels. We construct curricula to follow standardized accreditation and program evaluation expectations to ensure that our educational mandates and competency expectations are met. Via these standardized structures, we aim to consistently graduate physicians who are ready for unsupervised practice regardless of where they trained. We monitor learner performance to ensure that pre-determined competency expectations are met. Clearly, standardization helps ensure that physicians *do no harm*.

## The perils of standardization

But in complex situations, rigid adherence to standardized rules can be perilous. For instance, standardization can impede physicians’ ability to wield their expertise. Research tells us that novices tend to rigidly adhere to rules and guidelines because they lack the savvy to engage in the complex interpretations needed to exercise discretionary judgment [7]. In contrast, experts transcend reliance on rules because they have deep understanding and rich experiences through which to interpret each unique situation [7]. Physicians who harness their expertise don’t blindly adhere to standardization (e.g., CPGs). To be clear: we contend that guidelines are useful when a patient presents with, for example, typical asthma. But we also contend that when an expert physician senses that what is presenting as a *horse* is actually a *zebra*, we want them to be empowered to deviate from standard protocol. This is why standardization cannot be universally imposed, especially among expert clinicians.

Another threat posed by standardization lies in the structures that underpin medical education’s processes and curricula; they are perilous because they make responding to our ever-changing social context desperately difficult. For example, as Bates et al have argued, [8] medical schools’ use of standardized processes in admissions (e.g., MCAT scores) that aim to promote fairness can constrain the diversity of learners admitted to medical schools. So while our medical schools increasingly advocate for increasing the diversity of learners matriculating into their programs, the standardization of their admission processes risks undermining those very efforts. Here again, the standardization that is meant to achieve one social goal (i.e., fairness realized via the consistent use of standardized tests), is simulanteously eroding a similar and affiliated social goal (i.e., equity).

And so, we must wrestle with a paradox: standardization requires adherence to pre-determined tenets, but that adherence can undermine the very purposes of standardization. Therefore, as has long been implicitly understood but rarely explicitly acknowledged, we need to uphold standardization with dexterity—both relying on it and eschewing it as appropriate.

## The promise of adaptability

Achieving this dexterity between standardization and adaptability is possible when we embrace practice and education frameworks that support adaptable practices (e.g., enabling physicians to customize practices based on patient circumstances and their expertise). In other words, we can bridge the standardization-adaptability divide by practicing *adaptive standardization*—i.e., the capacity to apply protocols in ways that accommodate specificity, to pivot within frameworks to maintain functionality in specific situations [9].

Adaptive standardization can only be achieved when both individual physicians and the systems in which they work embrace this orientation. For instance, in medical education assessment, equity is often pursued through standardized, impartial evaluation practices. Yet excessive adherence to these standards can unintentionally perpetuate inequities. Kakara Anderson et al [10] describe this scenario by contrasting fairness-oriented assessment—which assumes assessments should be impartial without regard to social or cultural characteristics—with inclusion- and justice-oriented approaches, which inherently advocate for adaptability to accommodate diverse learners. They highlight how rigid assessment frameworks, while designed to ensure fairness and reduce bias, may fail to recognize varied learner contexts or effectively address deeper structural inequities. By embracing adaptive standardization—balancing consistent standards with context-sensitive flexibility—medical education can uphold fairness without sacrificing responsiveness to evolving societal demands and learner-specific needs.

## Why adaptability is so hard to embrace

Hospital management structures and medical education policies often depict standardization as safeguarding consistent delivery of high quality and efficient patient care—both today and in the years to come. But myopic focus on adherence to standardization can narrow hospital leaders’ focus, creating unfair scrutiny of clinicians and a fixed mentality that discourages nuanced judgement. And when this mentality reaches into the medical education system, we risk promoting complaceny and a one-size-fits-all approach in trainees whose practice reality will be far from stable.

Making room for more adaptability will require adjusting our view on standardization. It will require the hard work of thinking through each clinical and educational situation for exactly what it is—unique. If we could adopt an attitude that tempers standardization with adaptability, then we might find a space where we can reap the benefits of standardized frameworks while simultaneously allowing for adaptability to respond to social changes.
